# Privacy Risk Assessment of Smart Home System Based on a STPA–FMEA Method

**DOI:** 10.3390/s23104664

**Published:** 2023-05-11

**Authors:** Yue Wang, Rui Zhang, Xiaoyi Zhang, Yalan Zhang

**Affiliations:** School of Management, China University of Mining and Technology (Beijing), Beijing 100083, China

**Keywords:** smart home, interaction control risk, privacy risk, risk assessment, STPA, FMEA

## Abstract

Although the smart home industry is rapidly emerging, it faces the risk of privacy security that cannot be neglected. As this industry now has a complex combination system involving multiple subjects, it is difficult for the traditional risk assessment method to meet these new security requirements. In this study, a privacy risk assessment method based on the combination of system theoretic process analysis–failure mode and effect analysis (STPA–FMEA) is proposed for a smart home system, considering the interaction and control of ‘user-environment-smart home product’. A total of 35 privacy risk scenarios of ‘component-threat-failure-model-incident’ combinations are identified. The risk priority numbers (RPN) was used to quantitatively assess the level of risk for each risk scenario and the role of user and environmental factors in influencing the risk. According to the results, the privacy management ability of users and the security state of the environment have significant effects on the quantified values of the privacy risks of smart home systems. The STPA–FMEA method can identify the privacy risk scenarios of a smart home system and the insecurity constraints in the hierarchical control structure of the system in a relatively comprehensive manner. Additionally, the proposed risk control measures based on the STPA–FMEA analysis can effectively reduce the privacy risk of the smart home system. The risk assessment method proposed in this study can be widely applied to the field of risk research of complex systems, and this study can contribute to the improvement of privacy security of smart home systems.

## 1. Introduction

With the rapid development of big data, artificial intelligence, and 5G communication technology, people are increasingly becoming capable of mining and processing large volumes of data in the physical information world, and society is advancing towards the use the Internet of Things (IoT). Various applications derived from the IoT are being increasingly used, with smart home systems rapidly emerging as an important component of it. Smart home systems change the service mode of traditional home appliances and connect various devices (household appliances, communication devices, temperature control devices, security devices, etc.) within the home to a centralised control system. In recent years, the scale of the smart home market has expanded. The International Data Corporation (IDC) reports that global smart home device shipments will exceed 800 million units by 2020 and are expected to exceed 1.4 billion units by 2025; China’s smart home device market shipments will exceed 200 million units by 2020 and are expected to exceed 450 million units by 2025 [1].

However, smart homes have faced increasing security concerns while growing at a rapid pace. The IoT technology provides opportunities but comes along with risks. The smart home is based on the IoT but needs to meet higher security requirements because it contains critically important and sensitive private information. Smart home systems are vulnerable to cyber attacks, and if hacked, they are likely to lead to privacy leakage and even real-time monitoring of users [2]. Many potential consumers also give up using smart home systems because of their lack of trust in privacy and security measures [3]. Therefore, it is necessary to pay attention to privacy risk as a security issue in the development of smart homes.

Currently, companies often use a failure mode and effects analysis (FMEA)-based approach to analyse product quality and safety. This is a bottom-up method that predicts the possible failure of a device by analysing the potential failure modes and effects of product components, thus preventing accidents at the source. This method is widely used in aerospace [4], medical services [5], manufacturing production [6], and other fields. However, the traditional FMEA method has certain shortcomings; it tends to ignore the user’s risk in the FMEA phase, and does not consider the influence of user and environmental factors on the risk in the use scenario. Additionally, this method focuses on the assessment of product risk without a risk control component [7].

Leverson [8] proposed a risk control approach; systems theoretic process analysis (STPA) based on the systems theoretic accident model and process (STAMP). The STPA approach considers that system safety can be regarded as a control problem, and that system accidents are caused by uncontrolled interactions between participants. This approach provides a new research perspective on risk-control questions. Bensaci et al. [9] adapted the STPA approach to multi-intelligent systems with strong interactions, evaluated the system risk under different control methods, helped deciders identify low-risk system control methods, and verified the effectiveness of the STPA approach in the risk analysis of complex systems. Shapiro [10] applied the STPA method to security analysis of privacy engineering, identified privacy risks missed by traditional risk analysis methods, and proved that the STPA method can be used as a supplement to traditional risk analysis methods.

Smart home systems are multi-level complex systems that include sensing devices, control terminals, transmission networks, and actuation devices, which are closely related to user and environmental interactions [11]. In the security analysis of smart home system privacy, the potential risks that may exist in the system components and the risks caused by unsafe interactions among the product, user, and the environment should be evaluated [12,13]. In the already existing studies on smart home risks, only qualitative analysis of risk scenarios is usually carried out and few studies have quantified risks. However, only finding the key risk control points can improve the efficiency of risk control, which requires us to assess the risks quantitatively [14]. Among the commonly used quantitative risk assessment methods, the FMEA method is very widely used. Based on its characteristics of predicting risks in advance, emphasising the sources of risks and generality, this study selects the FMEA method for the assessment of privacy risks in smart homes. However, the FMEA method mainly focuses on the defects of components and it is difficult to assess the interaction and control effects between different subjects of the system. Therefore, this study integrates the STPA method into the FMEA method to construct the STPA–FMEA risk assessment framework, which can inherit the advantages of both. First, it can identify potential risks before design and process implementation, thus helping companies to identify preventive measures to reduce costs and risks. Second, it can help to identify defects in components themselves and unsafe interactions with users, the environment and the smart home system. Third, it can identify measures to reduce privacy risks in the smart home from a system control perspective. In addition, sinc the risk priority number (RPN) is one of the cores of the FMEA methodology, this study also chose the RPN value to quantitatively assess the privacy risk of smart home systems. The calculation of the RPN value requires three risk indicator values, which are severity, occurrence, and detection. Experts in the field of smart home are experienced and able to assess the three indicators more accurately to determine the risk factor for each risk scenario.

This study explores the privacy risk assessment of smart home systems, with four main contributions. First, this study constructs a STPA–FMEA risk assessment framework to assess the risks caused by component defects and insecure interaction control behaviours of smart home systems. Second, this study developed a four-level complex metric system for privacy risk in smart home systems as ‘component-threat-failure model-incident’. Third, this study assessed the role of user behaviour and environmental status in influencing privacy risk in the smart home system. Fourth, STAMP is applied to identify the insecure control behaviour of the ‘user-environment-smart home product’ and propose corresponding control measures. These contributions can help to control the privacy risk of smart home systems more comprehensively and accurately, and will be of some significance to improve the privacy security of the smart home system.

The rest of the paper is structured as follows: in Section 2, we introduce the literature review; in Section 3, we introduce the research methodology, and develop the risk assessment framework of STPA–FMEA, and establish the hierarchical control structure of ‘user-environment-smart home system’; in Section 4, we conduct the identification of privacy risks in smart home systems by developing a four-level complex indicator system; in Section 5, we assess the role of user and environment on each privacy risk value; in Section 6, measures to improve the privacy security of the smart home system are proposed; and in Section 7, we draw conclusions and outline the strengths of the findings of this study compared to previous studies.

## 2. Literature Review

### 2.1. Privacy Risk of Smart Home

As smart home products are usually used within the confines of the home, they have access to a large amount of users’ private information, making it easy for privacy risks to occur. To improve the privacy security of smart home systems, a large number of scholars have conducted research in this area. Charlie et al. [15] found that reducing the autonomy and independence of the home and increasing technological controls were most likely to create privacy risks, through a national survey of representative users of smart homes in the UK. Ni JB et al. [16] introduced a fog computing program to enhance IoT functionality, and the study found that fog computing has new requirements for system privacy and security. Additionally, addressing privacy issues still requires more attention and effort. Norman and Ksenia [17] constructed a framework for a unified theory of acceptance and use of technology that incorporates the structure of the privacy algorithm, and found that the issue of perceived privacy has a significant impact on perceived value. Francesca et al. [18] believe that many low-end IoT devices cannot support advanced security defence mechanisms, leading them to be targeted and even exploited by security attacks. The configuration parameters of most smart home devices are at a low level, which is already a weak point in cyber security, and coupled with the fact that they contain a lot of personal information, they are even more of a target.

### 2.2. Related Work

To address the issue of privacy risk in the smart home system, many scholars have conducted research from different perspectives, mainly including identifying organisational privacy threats, determining mitigation strategies, and assessing privacy risks. Specific research efforts are described below.

Tom et al. [19] had constructed a risk assessment model based on cloud services that encompasses security, privacy and resource management, in the context of integrated device management in the home. Key indicators, such as trust, risk, eco-efficiency and cost, are also introduced into the model to assess the risk of home resources in a more comprehensive way. However, this risk assessment model can only be used for special cases and is difficult to be widely applied to other smart home systems.

Jacobsson et al. [20] used the ISRA methodology to analyse the risks in the development of smart home automation systems, through an extensive survey of leading companies in the smart home industry, and found that the events with the highest level of risk were usually related to human factors or system components. Based on the nature of the human factor, the associated risks require complex treatment. This study provides meaningful conclusions, but the focus of the research is not on developing a generic framework for risk analysis.

Nurse et al. [21] developed a model framework for managing security and privacy risks in smart home systems based on user and technology factors in order to address these risks. The contribution of this study is that it simplifies the risk assessment methodology and allows more consumers interested in security and privacy risk management to use this framework and participate in smart home risk management. However, this study does not consider the vulnerabilities of different devices, so the framework still has strong limitations.

Psychoula et al. [22] constructed a framework for privacy risk assessment and management of environmental assisted living technologies for smart home systems. By analysing privacy-impacting features in smart homes, characteristic indicators for evaluating privacy risks were identified, enabling quantification of potential privacy risks. The usability of the framework was determined after testing in a real smart home environment. However, no privacy attacks were simulated in the testing environment, and neither any cyber attacks were considered.

To test the security of the Internet of Things in smart cities, Krichen et al. [23] proposed a framework built on two formalisms, attack trees and price timing, combined with the execution test language TTCN-3, to build a cloud-oriented architecture capable of ensuring the execution of tests and generating determinations.

Sturgess et al. [24] analysed the reasons leading to the complexity of privacy risks in smart home systems and came up with the three most important factors, the highly heterogeneous nature of smart appliances, the diversity of threat forms and the difficulty of assessing personal information. A capability-oriented, system-independent privacy risk assessment model is developed to address these issues. The model is well suited to the rapidly evolving smart home, but only allows for risk assessment and makes it difficult to disadvantage risk control points.

Park et al. [25] used a spatial network approach to analyse the IoT security threats in smart home systems that may lead to privacy breaches. An assessment framework was also constructed for social damage risk to measure the potential risk of IoT devices based on security scenarios that may occur in the home. The framework obtained from this study is useful for assessing device risk, but its dependence on a number of factors limits its generalisability.

Compared with related works, the STPA–FMEA risk assessment framework adopted in this study is able to analyse risk from both the perspective of the smart home system components themselves and the system control behaviour, which theoretically allows for a more comprehensive risk causation. In addition, this study also proposes risk control measures that can be used as a reference for a company’s decision-making. The framework proposed in this study is also generic enough to be applied to most complex system risk analysis work.

## 3. Research Methodology

In the STPA–FMEA approach, incidents are viewed as caused by the presence of insecure controls or constraints at various levels of the control structure during the system development and operation phases. This means that the potential failure modes, the effects of system components, and the interactions of user-product-environment control effects should be considered, while assessing the privacy risk of smart home systems using the STPA–FMEA method. Based on the basic process of the FMEA method, this study introduces the STPA theory at each stage of risk assessment to provide a more comprehensive and improved assessment of system privacy risk. A specific process for risk assessment was developed, according to the privacy risk characteristics of smart home systems, by integrating risk management frameworks (risk management principles and guidelines) into the STPA–FMEA method.

**Figure 1:**
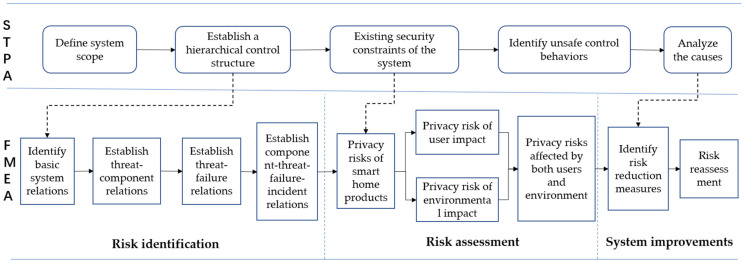
Framework for privacy risk assessment of smart home systems.

Figure 1 shows that the smart home system privacy risk assessment framework is divided into two directions: vertical, including the FMEA method and STPA method; and horizontal, including the three stages of risk identification, risk assessment, and system improvement. In the first stage, a hierarchical control structure is established based on the STPA method, and the smart home system structure is decomposed into basic constituent components based on the FMEA principles. Risk elements are identified through the correspondence of the component-threat-failure mode accident. In the second stage, the existing security constraints of the system are evaluated, and the changes in privacy risks under the roles of users and the environment are assessed. In the third stage, causative analysis of insecure control behaviours is performed, corresponding improvement measures for privacy security constraints of smart home systems are proposed, and risk reassessment is performed.

## 4. Risk Identification

### 4.1. Defining the Scope of the System

According to the standard GB/T41387-2022 and related literature [26,27,28], a smart home system is defined as a comprehensive system, including a smart home terminal, gateway, console, communication network, application service platform, and others. Because the impact of environmental and user factors on privacy risk in the usage of smart home system cannot be ignored, the research scope of this study is defined as the combined system of a ‘user-environment-smart home system’.

### 4.2. Establishing a Hierarchical Control Structure

**Figure 2:**
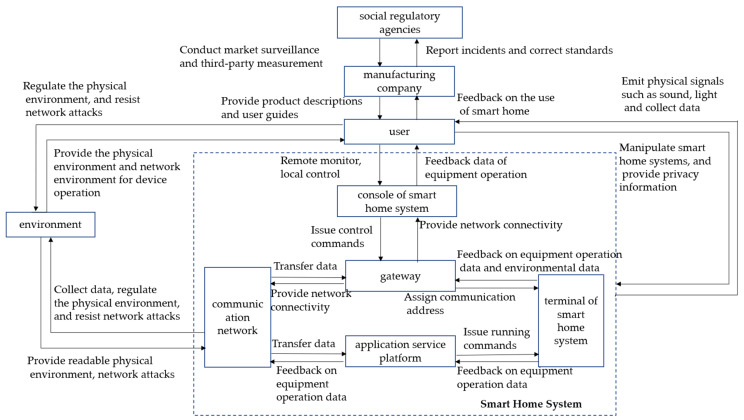
Smart home system hierarchical control structure.

In the smart home system risk assessment framework, a system hierarchical control structure must be identified as the basis for the FMEA expert to identify risk elements before performing risk identification. A hierarchical control structure is a system model consisting of control, feedback loops, and an effective control structure that allows constraints to be imposed on all the control behaviours of the system. These constraint–control behaviours may not be fully implemented during the development or use stage of the system, resulting in damage to the entire control structure or even an accident. The interaction between the user, the environment and the smart home system jointly determines the probability of privacy risks and the severity of the consequences. Therefore, each analysis module should include both the user and the environment. The smart home system usually consists of three levels: the control layer, the execution layer and the sensing layer, and the role relationships between the various components should be reflected in the layered structure. According to smart home related literature [20,21,22] and the Consumer Product Safety Risk Assessment Guidelines (GBT 22760-2020), the hierarchical control structure of the system was determined after discussions with experts, as shown in Figure 2.

Each analysis module of the hierarchical control structure consists of factors, such as users, environment, and products. The smart home system contains five parts: a smart home terminal, gateway, console, communication network, and an application service platform. The smart home terminal is a device that specifically realises the system function and collects environmental data for the user while completing control tasks from the user. The smart home gateway connects the smart home terminal, console, and application service platform, assigns communication addresses for each part, and provides a network connection. The smart home console is a device that interacts directly with the user, can receive commands from the user, and can pass commands downward while reflecting equipment operation data to the user. The smart home communication network includes a local area network and a wide area network that provides data communication services for the smart home system. The application service platform is an entity that supports the smart home system to realise the service and cooperates with the smart home console and terminal to realise the function.

Miandashti et al. [29] proposed that privacy risks in smart home systems mainly originate from the interactions between people, products, and the environment, secondary to the risks existing within the product components. When identifying specific risk scenarios, the user and environmental factors must be considered. According to the hierarchical control structure of the ‘user-environment-smart home’, the privacy and security risk characteristics are separately analysed from the perspective of the user, product, and environment to provide the basis for subsequent risk analysis.

(1) User Risk Characteristics

Jacobsson et al. [20] found that human behaviour is the most vulnerable risk factor for serious consequences in smart home systems. The risk features of users include the following three aspects: (1) In simple password settings, some users usually choose consecutive or inverted numbers as passwords due to their personal habits or poor memory, rendering it extremely easy to circumvent the identity verification mechanism, and providing hackers’ direct access to the system. (2) Due to the ease of trusting web information, gullible users are prone to fall into the traps set by hackers, resulting in information leakage and even property loss. (3) The concept of private security is weak; these users do not pay attention to protecting their private information when using the smart home application service platform, and the clues revealed are used in social engineering, leading to information leakage.

(2) Product Risk Characteristics

The sources of risk for smart home systems include system component failures and inadequate security control constraints at each system level. There is a risk of theft, damage, and manipulation of the system hardware with a moderate probability of occurrence but having serious consequences, and these factors are relatively easy to control. Improper configuration of system servers may cause risks, such as unauthorised modification of functions and data leakage. Inadequate constraints, such as access control and accountability mechanisms, can lead to hacking, resulting in significant loss of information and property [30].

(3) Environment Risk Characteristics

The risk characteristics of the environment typically include two aspects: one from the real environment, and the other from the network environment. There are frequent cases of malicious employees, third-party vendors, and others violating authorisation and illegally handling users’ personal information in the real environment. There are many types of cyberattacks in the network environment, such as phishing, blast attacks, and denial of service attacks. Different attacks have different targets, some of which start from luring users, and others directly attack devices or networks.

### 4.3. Identification of Risk Factors

Through the analysis of the hierarchical control structure, the existing security constraints, and control behaviours of the smart home system, as well as the risk features of the user, product, and environment, were clarified. Based on this, the Delphi method was used for the identification of risk factors, and 10 research members from this field were selected to form an expert group, hailing from universities, smart home manufacturing units, and standards institutes. Three rounds of interviews were conducted in this study, and the guiding topics in the first round of interviews were derived from system test data, and accident case data. The guiding topics in the next two rounds of interviews were derived from the collation and analysis of the expert opinions.

#### 4.3.1. Sources of Risk

The scope of the system defined in this study includes the users, the product, and the environment in which it is located. Under the principles of FMEA, a smart home system is the primary source of risk, and its components are secondary sources of risk. According to the structure of a smart home information system, system components are divided into four major categories: hardware (appliances, network devices, sensors, mobile devices, etc.), software (operating systems, applications, etc.), communication (network protocols, network technologies, communication endpoints, etc.), and information (data, etc.).

#### 4.3.2. Identification of Risk Events

In accordance with smart home appliances, personal information protection requirements, measurement methods GB/T40979-2021, and privacy risk-related literature [30,31], the expert group discussed and listed 17 main potential threats to the privacy security of smart home systems. These are as follows: insufficient physical protection mechanism, firmware logic vulnerability, lack of side letter channel protection, radio signal capture, insufficient authentication, inadequate accountability, entity–application interaction vulnerability, buffer overflow, programming error, malware, sensor and internal gateway inapplicability, API network protocol vulnerabilities, data poisoning, model inversion, third-party propagation, retransmission, and disorder.

By unifying expert opinions to determine the correlation between system privacy security vulnerabilities and smart home system components, the hazard variables identified as ‘component-threat’ are as follows:

A. Threats in hardware: insufficient physical protection mechanism, firmware logic vulnerability, lack of side-letter channel protection, and radio signal capture.

B. Threats in software: insufficient authentication, inadequate accountability, entity–application interaction vulnerability, buffer overflow, programming error, and malware.

C. Threats in communications: insufficient authentication, inadequate accountability, sensor and internal gateway inapplicability, and API network protocol vulnerabilities.

D. Threats in data: data poisoning, model inversion, third-party propagation, retransmission, and disorder.

To identify risk events that may result from risk sources, the associated potential hazard variables of the smart home system must be combined with the existing failure modes. Using the STPA–FMEA approach to study smart home failure modes requires a basis for smart home system components and security constraints. The failure modes of smart home systems are divided into three types based on expert opinions that are collated and analysed. First, physical security failure, which is the basis of device security, mainly includes physical entity security and the ability to fight network security attacks. Physical security failures are prone to device theft, node cloning, loss, and other defects. The second is control mechanism failure, which is the control failure in the use phase caused by the lack of automated detection and lack of effective application function testing in the development phase. The third is technical system failure, caused by the wrong design or inefficient technology of the product system, leading to problems such as arithmetic errors, delays, and collection deviations.

Similarly, the possible ‘component-threat-failure-mode’ risk events resulting from the risk source are identified based on the association of the hazard variables with the failure mode, as follows:

A. Physical security failures in hardware systems result in insufficient physical protection mechanisms, radio signal capture, control mechanism failures resulting in firmware logic vulnerability, and a lack of side letter channel protection.

B. Control mechanism failures in software systems lead to insufficient authentication, inadequate accountability, and system technical faults leading to entity–application interaction vulnerability, buffer overflow, programming error, and malware.

C. Control mechanism failures in communication systems result in insufficient authentication and accountability, system technology failures result in sensor and internal gateway inapplicability, and API network protocol vulnerabilities.

D. System technical faults in the data component lead to data poisoning, model inversion, third-party propagation, retransmission, and disorder.

#### 4.3.3. Risk Identification Results

If privacy risk events occur in smart home systems, they will have serious consequences for users, enterprises, and society. According to the smart home privacy violation cases released by China News and related literature [32,33], 14 types of incidents that could be caused by smart home privacy risk events were screened: changing system configuration, modifying device functions, remotely manipulating terminals, manipulating sensor configuration measurements, inferring user activities, controlling communication, learning user actions, loss, cloning, stealing, monitoring user residences, location tracking, information leakage, and access to classified data. Based on the correlation between risk event causes and accident variables, a combination of risk events and accidents is identified, which is a risk scenario consisting of ‘component-threat-failure mode-accident’. The identification results are listed in a five-level index system: the first-level index consists of users, the smart home system, and the environment; the second-level index consists of four types of components of the smart home system; the third-level index consists of 17 potential threats to the smart home system; the fourth-level index consists of three failure modes of the smart home system; and the fifth-level index consists of 14 possible accidents caused by risk events. Specific risk identification results are presented in Table 1.

## 5. Risk Assessment

The risk identification phase identified 35 privacy risk scenarios with the ‘component-threat-failure mode-incident’ combination, followed by a quantitative assessment of the risk values for each risk scenario using the RPN algorithm of the FMEA method. In addition to following the classical FMEA scoring principles, the evaluation process also considers the ‘user-environment-smart home’ hierarchical control structure of the new approach to estimate the impact of user and environment on risk values.

### 5.1. Quantitative Analysis of Risk

In the STPA–FMEA method, the RPN value was used to quantitatively assess the privacy risk of the smart home system, and the RPN values of 35 risk scenarios were calculated separately to assess the risk degree of each scenario. RPN is obtained by the multiplication of the severity of consequences (*S*), the probability of occurrence (*O*), and the detectability (*D*) of risk scenarios, that is, RPN = *S* × *O* × *D*. The larger the value, the higher is the potential risk. Additionally, the SOD scoring in this study not only scores the product risk alone but also considers the interactive control of humans, machines, and the environment, which adds to the assessment of user and environment impact on the system privacy risk. Therefore, the user influence coefficient (*UC*) and environmental influence coefficient (*EC*) are introduced to improve the traditional RPN. Furthermore, *UC* represents the effect of the user’s privacy management capability on a specific privacy risk event on the RPN value, defined as the product of the user’s privacy management capability and the extent of the user’s effect on the risk. Additionally, *EC* represents the effect of environmental conditions on the RPN value of a specific privacy risk event, defined as the product of environmental assessment value and the extent of the environmental effect on the risk of the product of environmental assessment value and the extent of the environmental influence on the risk. The improved RPN value calculation using Equation (1) is as follows:

(1) $$\mathrm{RPN}=(S\times UC_{S}\times EC_{S})\times (O\times UC_{O}\times EC_{O})\times (D\times UC_{D}\times EC_{D})$$

where, *UC_S_* denotes the user impact coefficient for consequence severity (*S*) and *EC_S_* denotes the environmental impact coefficient for consequence severity (*S*). Furthermore, *UC_O_* denotes the user impact coefficient for the event occurrence probability (*O*), and *EC_O_* denotes the environmental impact coefficient for the event occurrence probability (*O*). Additionally, *UC_D_* denotes the user impact coefficient for failure detectability (*D*) and *EC_D_* denotes the environmental impact coefficient for failure detectability (*D*).

#### 5.1.1. User Factor

As the direct controller of the smart home system, the user’s behaviour has a significant effect on the probability of privacy risk events and the severity of the consequences of the smart home system. The user’s education level, cognitive ability, judgment ability, personality characteristics, and occupation type all exert a certain influence on the user’s behaviour. To quantitatively evaluate users’ privacy management ability, a questionnaire survey and fuzzy comprehensive evaluation method were used for the analysis.

The comprehensive evaluation index of the user privacy management ability is U = {privacy awareness, judgment ability, prevention awareness, and data management ability}, and the weights of each index were assigned equally. The set of comments was V = {very strong, strong, average, weak, and very weak}, and the corresponding assigned values were {0.2, 0.5, 1, 2, and 5}. The following representative type of consumers were selected to distribute the questionnaire: (a) Age 12–16/education: junior high school and below, (b) Age 17–28/education: high school or specialist, (c) Age 29–45/education: bachelor and above, and d. Age 46–68/education: specialist and below. Collating the questionnaire results, the fuzzy relationship matrix of user ability for each consumer type was obtained as follows:

$$R_{a}=\left[\begin{array}{ccccc} 0.1 & 0.3 & 0.3 & 0.2 & 0.1\\ 0 & 0.1 & 0.4 & 0.3 & 0.2\\ 0.1 & 0.2 & 0.2 & 0.3 & 0.2\\ 0 & 0.1 & 0.3 & 0.2 & 0.4 \end{array}\right]R_{b}=\left[\begin{array}{ccccc} 0.4 & 0.3 & 0.2 & 0.1 & 0\\ 0.2 & 0.3 & 0.4 & 0.3 & 0\\ 0.2 & 0.3 & 0.3 & 0.2 & 0\\ 0.3 & 0.3 & 0.2 & 0.1 & 0.1 \end{array}\right]\;R_{c}=\left[\begin{array}{ccccc} 0.1 & 0.3 & 0.3 & 0.3 & 0\\ 0.3 & 0.3 & 0.2 & 0.2 & 0\\ 0.3 & 0.3 & 0.3 & 0.1 & 0\\ 0.1 & 0.2 & 0.4 & 0.2 & 0.1 \end{array}\right]R_{d}=\left[\begin{array}{ccccc} 0.1 & 0.3 & 0.4 & 0.1 & 0.1\\ 0.1 & 0.1 & 0.5 & 0.2 & 0.1\\ 0.1 & 0.2 & 0.4 & 0.2 & 0.1\\ 0.1 & 0.1 & 0.5 & 0.1 & 0.2 \end{array}\right]$$

The factor weight set and fuzzy relationship matrix were fuzzily transformed according to model M (−, +).

$$B=\left[\begin{array}{ccccc} 0.05 & 0.175 & 0.3 & 0.25 & 0.225\\ 0.275 & 0.3 & 0.225 & 0.175 & 0.025\\ 0.2 & 0.275 & 0.3 & 0.2 & 0.025\\ 0.1 & 0.175 & 0.45 & 0.15 & 0.125 \end{array}\right]$$

Finally, the data at each indicator level were normalised to calculate the assessed values of privacy management capabilities for the four typical consumer types based on the assignment of each element of the rubric set, and the results were as follows.

$$\left(\begin{array}{cccc} a & b & c & d \end{array}\right)=\left(\begin{array}{cccc} 2.023 & 0.905 & 1.003 & 1.483 \end{array}\right)$$

#### 5.1.2. Environmental Factors

The security of the environment in which the smart home system is located directly determines the possibility of being attacked, thus influencing the system privacy risk. Environmental factors include the physical, network, and social environments. The environmental assessment value is quantified according to the level of security, which is assigned as {0.2, 0.5, 1, 2, and 5} according to whether it is very secure, more secure, average, less secure, or least secure.

#### 5.1.3. Risk Assessment Results

Expert groups were invited to assess the user and environmental impact values of privacy risks using interviews and questionnaires. The experts scoring the SOD considered the hierarchical control structure of the smart home system and referred to the FMEA Handbook (5th edition) scoring criteria. The scoring results of the ten experts were summarised and collated, and the average value was taken as the assessment result of privacy risk, as shown in Table 2.

The SOD scores for each risk scenario under the traditional FMEA approach and the SOD scores under the STPA–FMEA approach are presented in Table 2. The SOD scores include the original RPN scores for each risk scenario and the magnitude of the impact of user factors, physical network, and social environment on the risk.

### 5.2. Analysis of Risk Assessment Results

**Figure 3:**
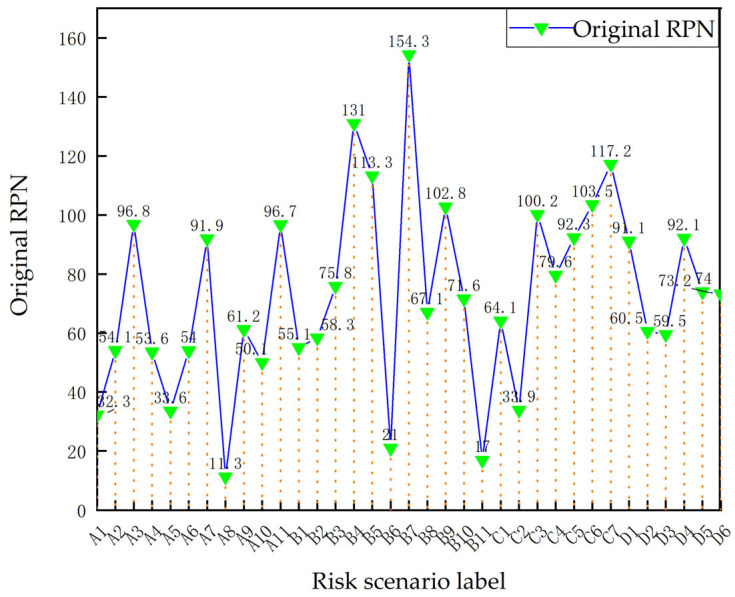
Original RPN for privacy risk of smart home system.

The RPN diagram of each risk scenario under the traditional FMEA method was drawn based on the privacy risk assessment data of the smart-home system, as shown in Figure 3. The five risk scenarios with the highest risk values in the smart home system obtained using the traditional FMEA method are as follows.

B7: In software systems, the entity–application interaction vulnerability caused by technical system faults may result in controlling communication.

B4: In software systems, inadequate accountability caused by control mechanism failures may result in modifying device functions.

C7: In communication systems, API network protocol vulnerabilities caused by technical system faults may result in cloning.

B5: In software systems, the entity–application interaction vulnerability caused by technical system faults may result in the remote manipulation of terminals.

C6: In communication systems, API network protocol vulnerabilities caused by technical system faults may result in losses.

**Figure 4:**
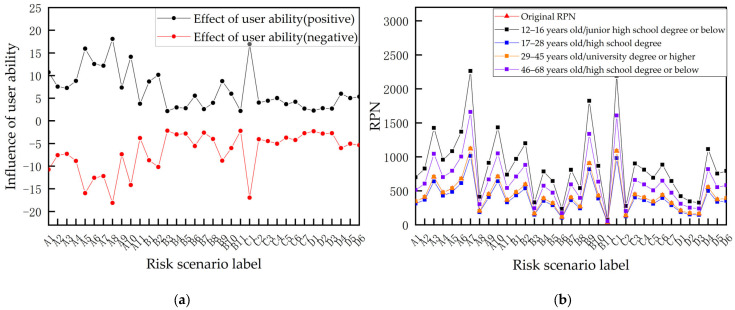
RPN of privacy risk under users’ effects. (**a**) Impact of user capabilities on RPN, (**b**) RPN for risk scenarios under different types of users. As shown in Figure 4a, the three risk scenarios in which user capabilities have the greatest impact on risk valuation are as follows: A5, in hardware systems, firmware logic vulnerability caused by physical security failures can result in losses. C1, in communication systems, insufficient authentication caused by control mechanism failures can result in remotely manipulating terminals. A8, in hardware systems, the lack of side-letter channel protection caused by physical security failures can result in losses. As shown in Figure 4b, the risk values of the four categories of users differ significantly under different risk scenarios, meaning that users of different ages and education levels have a significant impact on the risk profile. Users with higher privacy management capability (age 29–45, education bachelor’s degree or above) face lower privacy risk when using a smart home, but users with lower capability (age 12–16, education junior high school or below) face higher privacy risk.

**Figure 5:**
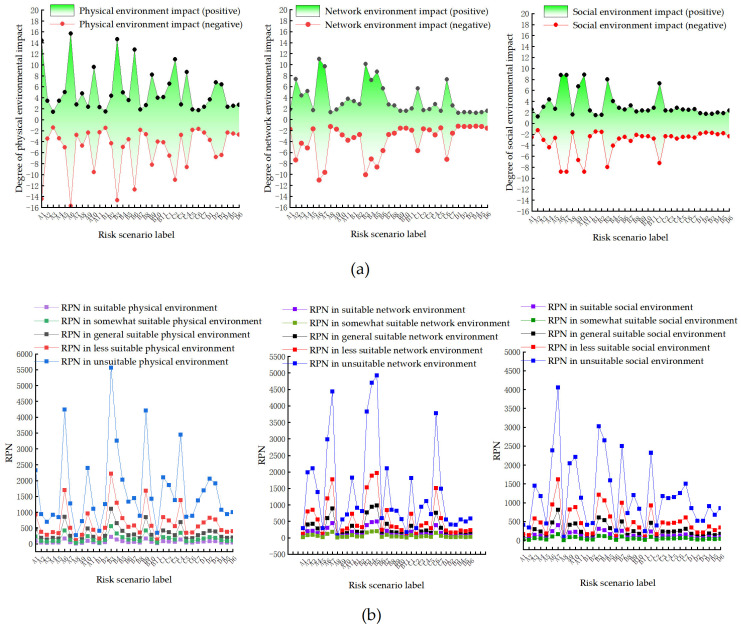
RPN of privacy risk under environmental effects. (**a**) Impact of environment on RPN, (**b**) RPN for risk scenarios in different environments.

To illustrate the interactive control role of the user and environment in the smart home system, RPN diagrams of risk scenarios under the influence of user capability (shown in Figure 4) and risk scenarios under the influence of different environmental factors (shown in Figure 5) were drawn according to the STPA–FMEA evaluation method, showing the level of influence of user capability and environmental conditions on the privacy risk of the smart home system.

As shown in Figure 5a, the three risk scenarios in which the physical environment has the greatest impact on risk valuation are as follows. A6, in hardware systems, the lack of side-letter channel protection caused by physical security failures may result in losses. B3, in software systems, inadequate accountability caused by control mechanism failures may result in the inference of user activities. A1, in hardware systems, insufficient physical protection mechanisms due to physical security failures may result in stealing. The three risk scenarios in which the network environment has the greatest impact on risk valuation are A6, B3, and A7. The three risk scenarios in which the social environment has the greatest impact on risk valuation are A10, A7, and A6. As shown in Figure 5b, the risk scenarios under the same environmental factors vary significantly in the value of the risk in different environmental states with different degrees of security. Changes in risk scenarios vary under different environmental factors corresponding to changes in environmental security. Among these, the network environment has the greatest impact on the privacy risk of a smart home system, whereas the physical and social environments have a secondary impact. The system risk decreases when the security of the environment increases and the system risk increases when the security of the environment decreases.

**Figure 6:**
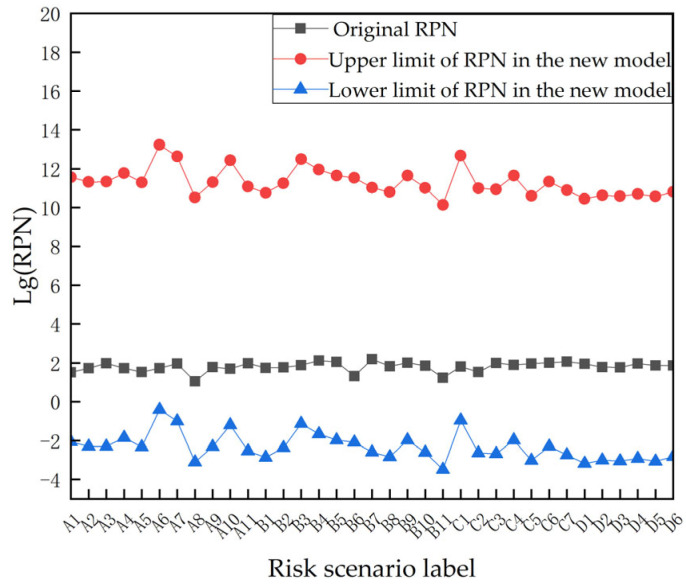
RPN under STPA–FMEA method.

As shown in Figure 6, the range of RPN variation for each risk scenario varies significantly in the risk assessment using the STPA–FMEA method. The difference in RPN values between the most advantageous user and the environmental conditions, and the most disadvantageous user and the environmental conditions, are huge, which shows that the impact of the user and environment on the risk of the smart home system cannot be underestimated. The five risk scenarios with the highest risk values under the new method are as follows:

A6: In hardware systems, a lack of side-letter channel protection caused by physical security failures can result in losses.

A7: In hardware systems, the lack of side-letter channel protection caused by physical security failures can result in location tracking.

C1: In communication systems, insufficient authentication caused by control mechanism failures can result in remote manipulation of terminals.

B3: In software systems, inadequate accountability caused by control mechanism failures can result in inference of user activities.

A10: In hardware systems, the lack of side-letter channel protection caused by control mechanism failures can result in location tracking.

Evidently, the privacy risk of the smart home system assessed using the STPA–FMEA method is completely different from the assessment result of the traditional FMEA method, which indicates that user and environmental factors have a significant impact on the private security of the smart home system. The traditional FMEA method ignores the interactive control role of both the smart home systems and only considers the smart home product itself as the risk assessment object, which has certain limitations in the assessment results. The STPA–FMEA method can be used to obtain relatively accurate and reasonable risk assessment results by taking a more comprehensive perspective and combining the interaction of various parts of a ‘user-environment-smart home’.

## 6. Improvement of the System

### 6.1. Control Measures for Risks

Taking the risk scenario with the highest risk value as an example, the STPA method was used to perform a causative analysis to take appropriate control measures. Following the STPA steps, after identifying system-level accidents and threats, unsafe control actions (UCA) must be identified based on the established system hierarchical control structure. The causative analysis of accidents can be performed through an analysis of the system control loops, and new safety constraints should be proposed to address the causes of accidents. Among them, UCA are usually classified into four categories: not providing the controlled act required by the system, providing the wrong control act, providing the controlled act too early or too late, and with too long or too short a time limit [34].

Based on the risk assessment results, the two scenarios with the highest risk values (A6 and A7) were selected for this study to perform risk control using the STPA method. It was determined that system-level incidents requiring analysis were the surveillance of user residences and user location tracking. Monitoring the user’s residence refers to a smart home system terminal being attacked and devices with audio-visual functions in the home being manipulated to monitor and eavesdrop on the residence, or the audio-visual information of the residence being illegally accessed due to illegal intrusion at other levels of the system. User location tracking refers to illegal access by an attacker after the user sends their location information to the location server.

To ensure the safe operation of the smart home system, it is necessary to analyse the control and feedback loops of the entire system control structure of the ‘user-environment-smart home’ to identify the unsafe control behaviours that may lead to hazards. According to the UCA classification in the STPA method, the unsafe control behaviours of the system are identified as follows. UCA1: no network security protection technology is provided; UCA2: no applicable security protection technology is provided; UCA3: no applicable security mechanism is provided; UCA4: security protection is provided too early or too late; and UCA5: security warning function is not enabled in real-time.

According to the STAMP theory, control defect classification [35] and the control structure of the smart home system, the causative factors were analysed in three aspects: control, feedback, and coordination defects. The causative factors are shown in Table 3.

The privacy risk control measures of the smart home system obtained based on the results of causation analysis are as follows: (a) Users must set high-strength passwords before entering the system; (b) Society strengthens the construction of NQI for the smart home system; (c) Enterprises provide users with instructional videos on the use of the smart home system, and users can enable the system only after passing the basic test; (d) Timely notifications of system updates and delayed change warnings must be sent; (e) Terminal-aware devices set up lightweight cryptographic algorithms and lightweight security authentication protocols; (f) The communication network layer establishes end-to-end and node-to-node encryption mechanisms and negotiation protocols; (g) Application service platform establishes reliable authentication mechanisms and key management schemes, improves intrusion detection and anti-virus detection capabilities, establishes malicious command analysis and prevention and access control, disaster recovery mechanisms, and establishes rebel tracking and other information leakage tracking mechanism; and h. Set up the way to physically switch off the audio-visual function of the device.

### 6.2. Evaluation of Risk Control Measures

Having identified the risk control measures, their effectiveness must be tested. Referring to the study by Yanxi and Tiezhong [36], a risk correction factor α was introduced to indicate the degree of influence of each constraint behaviour on the privacy risk of a smart home system. The impact of the new security constraints was assessed again by an expert group. Experts were asked to score the degree of risk impact of each control in the range of 0–1, in which scores closer to 0 indicated a higher degree of impact and scores closer to 1 indicated a lower degree of impact. The scoring results of the ten experts were aggregated and averaged, and the result was α = {a, b, c, d, e, f, g, h} = {0.14, 0.12, 0.28, 0.45, 0.25, 0.25, 0.2, 0.3}, respectively. Combining the risk correction factor α with Equation (1) results the new RPN calculation formula, as shown in Equation (2).

(2) $$\mathrm{RPN}=\alpha \times (S\times UC_{S}\times EC_{S})\times (O\times UC_{O}\times EC_{O})\times (D\times UC_{D}\times EC_{D})$$

**Figure 7:**
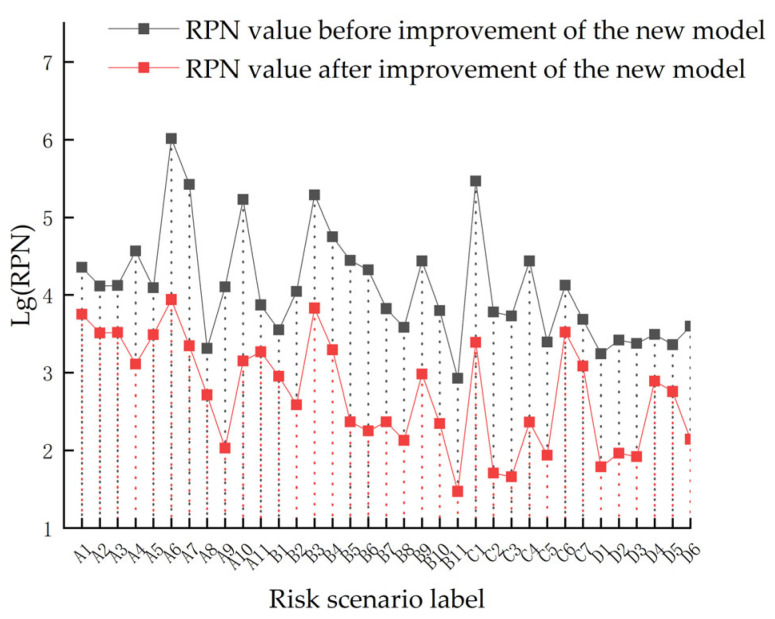
Privacy risk of the improved smart home system.

As shown in Figure 7, the RPN values of each privacy risk scenario of the smart home system are significantly reduced after the adoption of risk control measures. In particular, the RPN values of risk scenarios A6, A7, A9, A10, B5, B6, C1, C2, C3, and C4 show significant reduction. It can be seen that the control measures have a significant effect on reducing the system risk and can largely avoid the occurrence of accidents involving the surveillance of users’ residences, user location tracking, remote control of terminals, and modification of device functions.

## 7. Conclusions

In this study, the STPA–FMEA method was constructed by combining the traditional FMEA method with the STPA risk control method based on the system theory accident model and process. It was innovatively applied to the privacy risk assessment of smart home systems to identify the privacy risks in smart home usage scenarios, quantitatively assess the risk profiles of different privacy risk scenarios, and propose effective improvement measures.

Joseph [4] used the PRASH framework to analyse smart home privacy risks, but the risk scenarios identified and evaluated by the study were mainly due to cyber attacks. Reyhan [37] proposed measures to control privacy risks from the perspective of human factors only. In contrast, this study analyses human factors, environmental effects, component defects, and the privacy risks resulting from the interaction of the three. Privacy risks and possible vulnerabilities in the IoT have been analysed from multiple perspectives, but the evaluation of privacy risks are mostly qualitative and cannot measure the degree of risk in different damage scenarios. The risk assessment framework proposed in this study is able to quantitatively assess the risk using RPN values as well as the degree of influence of user and environmental factors on the risk [38]. Jacobsson et al. [20] identified privacy security needs through risk analysis and proposed corresponding risk control measures in the design phase. Based on the STAMP idea, this study identifies insecure behaviours throughout the control process and proposes control measures that should be taken by each subject separately. Therefore, the risk factors identified in this study are relatively very comprehensive, and the control measures proposed in this study are also relatively scientific and reasonable, contributing significantly to the reduction in privacy risks in smart home systems.

This study found that (1) using the STPA–FMEA method for privacy risk assessment of smart home systems comprehensively consider the interactive control role of the user, environment, and smart home, which obtains assessment results completely different from the traditional FMEA method and thus is more scientific and reasonable compared to traditional FMEA method. (2) User and environmental factors have a great influence on the privacy risk of smart home systems, and varying the privacy management ability of consumers using smart home products in different states of the environment can cause significant discrepancies in privacy risks. (3) The improvement measures proposed from the system control perspective using the STPA method have a significant effect on reducing the privacy risks of smart home systems.

The STPA–FMEA method used in this study performs well in the privacy risk assessment of smart home systems, and can be applied to the risk analysis of more complex systems to help managers obtain more reasonable and effective risk control measures to reduce system risk.

There are intricate correlations in the risk scenarios assessed in this paper, which are not explored further here. We will use a network approach to explore the correlation of risk factors in smart home systems in the future to propose more targeted privacy risk control measures.

## Figures and Tables

**Table 1:** Risk identification results.

Components for Smart Home System	Threats	Failure Modes	Incidents	Symbol
hardware systems	insufficient physical protection mechanism	physical security failures	stealing	A1
			loss	A2
			cloning	A3
	firmware logic vulnerability	physical security failures	modifying device functions	A4
			loss	A5
		control mechanism failures	monitoring user residence	A6
			location tracking	A7
	lack of side letter channel protection	physical security failures	loss	A8
		control mechanism failures	monitoring user residence	A9
			location tracking	A10
	radio signal capture	physical security failures	learning user actions	A11
software systems	insufficient authentication	control mechanism failures	changing system configuration	B1
			modifying device functions	B2
	inadequate accountability	control mechanism failures	inferring user activities	B3
			modifying device functions	B4
	entity–application interaction vulnerability,	system technical faults	remotely manipulating terminals	B5
			manipulating sensor changing system configuration measurements	B6
			controlling communication	B7
	buffer overflow	system technical faults	remotely manipulating terminals	B8
	programming error	system technical faults	remotely manipulating terminals	B9
			information leakage	B10
	malware	control mechanism failures	access to classified data	B11
communication systems	insufficient authentication	control mechanism failures	remotely manipulating terminals	C1
			manipulating sensor changing system configuration measurements	C2
	inadequate accountability	control mechanism failures	changing system configuration	C3
			modifying device functions	C4
	sensor and internal gateway inapplicability	control mechanism failures	controlling communication	C5
	API network protocol vulnerabilities	system technical faults	loss	C6
			cloning	C7
data component	data poisoning	system technical faults	information leakage	D1
	model inversion	system technical faults	access to classified data	D2
	third-party propagation	system technical faults	information leakage	D3
	retransmission	system technical faults	loss	D4
			cloning	D5
	disorder	system technical faults	loss	D6

**Table 2:** Results of risk assessment.

Risk Scenario Label	Severity *S*	Occurrence Rate *O*	Detectability *D*	Original RPN
A1	(2.3, 1.7, 2.5, 1.2, 1.2)	(1.8, 3.5, 1.8, 1.5, 2.1)	(7.8, 1.8, 3.2, 1, 1)	32.29
A2	(4.6, 1, 1.5, 2.3, 0.8)	(2.1, 3.6, 2.3, 3.2, 1.3)	(5.6, 2.1, 1, 1, 1.2)	54.10
A3	(7.2, 1, 1.3, 1.3, 1.3)	(2.8, 2.6, 1, 2.8, 2.1)	(4.8, 2.8, 1.1, 1.2, 1.1)	96.77
A4	(6.5, 1.2, 1, 1.9, 1.6)	(1.1, 3.2, 1.9, 2.1, 2.1)	(7.5, 2.3, 1.8, 1.3, 1.3)	53.63
A5	(2.3, 2.1, 1.2, 1.1, 1.2)	(3.4, 2.3, 2.1, 1.2, 1.7)	(4.3, 3.3, 2, 1.3, 1.3)	33.63
A6	(3.1, 1.5, 1.7, 1.5, 1.5)	(2.6, 2.2, 3.3, 2.3, 2.1)	(6.7, 3.8, 2.8, 3.2, 2.8)	54.00
A7	(3.8, 1.5, 1.1, 1.5, 1.5)	(3.9, 2.8, 2.1, 2.3, 2.8)	(6.2, 2.9, 1.2, 2.8, 2.1)	91.88
A8	(2.3, 1.9, 1.7, 1, 1)	(1.2, 3.4, 2.8, 1.2, 1.6)	(4.1, 2.8, 1, 1.1, 1)	11.32
A9	(5.2, 1, 1.3, 1.2, 1.2)	(2.1, 2.3, 1, 1.5, 3.1)	(5.6, 3.2, 1.8, 1, 1.8)	61.15
A10	(5.3, 1.3, 3.2, 1.8, 1.2)	(4.5, 3.4, 2.3, 1.2, 2.3)	(2.1, 3.2, 1.3, 1.3, 3.2)	50.09
A11	(3.2, 1, 1, 1.8, 1.3)	(5.7, 2.1, 1, 2.1, 1.8)	(5.3, 1.8, 2.3, 1, 1)	96.67
B1	(2.3, 2.3, 1, 1.2, 1)	(3.8, 1.8, 1, 1.2, 1)	(6.3, 2.1, 1.5, 2.3, 1.5)	55.06
B2	(2.2, 2.2, 1.2, 1.1, 1.2)	(3.9, 2.2, 1, 1.2, 1)	(6.8, 2.1, 3.6, 2.1, 1.3)	58.34
B3	(5.4, 1.2, 1.1, 2.1, 1.9)	(1.8, 1, 3.1, 3.2, 3.5)	(7.8, 1.8, 4.3, 1.5, 1.2)	75.82
B4	(5.3, 1.3, 1.2, 1.9, 1.5)	(3.8, 1.2, 1.8, 2.1, 1.8)	(6.5, 1.9, 2.3, 1.8, 1.5)	130.91
B5	(2.9, 1.3, 1.3, 2.3, 1.3)	(6.2, 1.2, 1.1, 2.1, 1.8)	(6.3, 1.8, 2.5, 1.8, 1.2)	113.27
B6	(1.5, 2.2, 1.3, 1.5, 1.1)	(3.1, 1.1, 3.5, 2.1, 1.2)	(4.5, 2.3, 2.8, 1.8, 1.9)	20.93
B7	(5.6, 1.2, 1.2, 1.3, 1.5)	(5.3, 1.2, 1.3, 1.5, 1.8)	(5.2, 1.8, 1.2, 1.4, 1.2)	154.34
B8	(4.7, 1.3, 1.3, 1.5, 1.5)	(2.8, 1.8, 1.2, 1.2, 1.1)	(5.1, 1.7, 1.7, 1.4, 1.3)	67.12
B9	(5.2, 1.9, 1.5, 1.2, 1.2)	(3.8, 2.1, 2.6, 1.1, 1.3)	(5.2, 2.2, 2.1, 1.2, 1.5)	102.75
B10	(3.2, 1.9, 1.4, 1.2, 1.2)	(5.2, 2.1, 1.5, 1.2, 1.5)	(4.3, 1.5, 1.9, 1.1, 1.3)	71.55
B11	(2.1, 1.2, 1.2, 1.2, 1.2)	(2.3, 1.3, 1.9, 1.5, 1.8)	(3.5, 1.4, 1.8, 1.1, 1.3)	16.91
C1	(3.1, 2.1, 1.3, 1.5, 1.5)	(3.9, 2.6, 1.8, 2.1, 2.1)	(5.3, 3.1, 2.8, 1.8, 2.3)	64.08
C2	(2.8, 1.5, 1.8, 1.2, 1.3)	(3.1, 1.5, 2.1, 1.1, 1.5)	(3.9, 1.8, 2.9, 1.3, 1.2)	33.85
C3	(6.5, 1.9, 1.2, 1.2, 1.2)	(2.3, 1.3, 1, 1.2, 1.5)	(6.7, 1.8, 2.3, 1.3, 1.3)	100.17
C4	(4.5, 2.1, 1.2, 1.3, 1.3)	(2.6, 1.6, 1.9, 1.8, 1.8)	(6.8, 1.5, 3.8, 1.2, 1.2)	79.56
C5	(3.8, 1.3, 1.1, 1, 1.2)	(4.5, 1.5, 1.2, 1.3, 1.6)	(5.4, 1.9, 1.4, 1.2, 1.3)	92.34
C6	(5.3, 1.1, 1.1, 1.2, 1.3)	(6.1, 1.2, 1.2, 3.2, 1.1)	(3.2, 3.2, 1.3, 1.9, 1.7)	103.46
C7	(6.3, 1.1, 1, 1.3, 1.1)	(6, 1.3, 1.8, 1.3, 1.8)	(3.1, 1.9, 1.3, 1.5, 1.3)	117.18
D1	(3.8, 1.3, 1.5, 1.1, 1.2)	(5.1, 1.1, 1.3, 1.1, 1.3)	(4.7, 1.6, 1.9, 1, 1.2)	91.09
D2	(3.2, 1.2, 1.8, 1, 1.1)	(4.2, 1.3, 2.1, 1.1, 1.3)	(4.5, 1.8, 1.8, 1.2, 1.2)	60.48
D3	(3.3, 1.1, 1.7, 1, 1.2)	(4.1, 1.3, 2.1, 1.1, 1.2)	(4.4, 1.9, 1.8, 1.2, 1.2)	59.53
D4	(5.1, 1.5, 1.5, 1.1, 1.1)	(4.3, 1.9, 1.3, 1, 1.5)	(4.2, 2.1, 1.2, 1.1, 1.2)	92.11
D5	(4.2, 1.4, 1.4, 1, 1)	(4.1, 1.8, 1.4, 1.1, 1.4)	(4.3, 2, 1.3, 1.2, 1.3)	74.05
D6	(3.8, 1.5, 1.5, 1.1, 1.3)	(4.7, 1.7, 1.3, 1.2, 1.5)	(4.1, 2.1, 1.4, 1.2, 1.2)	73.23

**Table 3:** Analysis of causes.

Defect Category	Causal Factors
Control defects	1–1 Users fail to set high-strength passwords, leaving the system vulnerable to intrusion.
	1–2 Completely safe NQI for smart home systems failed to be provided.
	1–3 System security is at high risk due to users’ misuse.
	1–4 Users do not update the system in time.
	1–5 No detection of DOS attacks is provided at the communication network layer.
	1–6 Detection of network attacks, intrusions, viruses, etc. is not provided in the application service platform.
Feedback defects	2–1 The feedback data from the terminal sensing device is incomplete.
	2–2 Perception data is not backed up in the application service platform.
	2–3 Perception data is not encrypted in the application service platform.
	2–4 Identity privacy protection and location privacy protection technologies are not provided in the application service platform.
Coordination defects	3–1 End-to-end and node-to-node encryption is not working.
	3–2 Communication network transmits wrong instructions and the feedback from the control terminal is not timely.
	3–3 User’s residence is monitored by terminal devices and the user does not take control.

## Data Availability

The data supporting the findings of this study are available from the corresponding author upon reasonable request.
